# Lower extremities task of pressing an “accelerator” or a “brake”: association with traffic accidents in older drivers – a preliminary study

**DOI:** 10.1590/1980-5764-dn-2022-0033

**Published:** 2022-11-04

**Authors:** Kenichi Meguro, Keiichi Kumai

**Affiliations:** 1 Tohoku University, New Industry Creation Hatchery Center, Geriatric Behavioral Neurology Project, Sendai, Japan.; 2 Tohoku University, Cyclotron Radioisotope Center, Sendai, Japan.; 3 Tohoku University, School of Medicine, Sendai, Japan.; 4 The Wakuya Medical and Welfare Center, Wakuya, Japan.

**Keywords:** Aging, Automobile Driving, Attention, Envelhecimento, Condução de Veículo, Atenção

## Abstract

Traffic accidents by older drivers are a social urgent problem. The National Police Agency (NPA) in Japan has institutionalized the *Cognitive Function Test* (NPA test) for renewal of a driver’s license for older adults. However, driving ability cannot be simply evaluated by usual cognitive tests on the desk.

**Objective:**

It is important to add an on-road test, but if not possible, we can use simulators. Before doing simulators, it is important to use the right foot to control the accelerator and brake pedals. We applied the Posner paradigm (visual attention test) for lower extremities.

**Methods:**

The participants were older adults. They and their families had anxiety about their driving. The 66 participants (44 men and 22 women) were divided into groups with and without experience of a traffic accident, and the following tests were examined: General cognitive and executive function tests, the NPA test, and an original Lower Extremity Reaction Test. Each participant was asked to press the “brake” or “accelerator” pedal by the right foot as quickly as possible in response to a traffic situation shown on the screen.

**Results:**

Compared to participants with favorable reactions to the Lower Extremity Reaction Test, those with poor reaction time tended to have more traffic accidents (OR=6.82), rather than the result of the NPA test.

**Conclusions:**

The results suggest that the probability of having a traffic accident can be better evaluated using the Lower Extremity Reaction Test.

## RESUMO

Os acidentes de trânsito por motoristas idosos são um problema social urgente. A Agência Nacional de Polícia (*National Police Agency –* NPA) no Japão institucionalizou o Teste de Função Cognitiva (teste NPA) para renovação de carteira de motorista para idosos. No entanto, a capacidade de dirigir não pode ser avaliada simplesmente por testes cognitivos usuais escritos.

**Objetivo:**

É importante adicionar um teste em estrada, mas se não for possível, simuladores podem ser utilizados. Antes de fazer simulações, é importante usar o pé direito para controlar os pedais do acelerador e do freio. Aplicamos o paradigma de Posner (teste de atenção visual) para extremidades inferiores.

**Métodos:**

Os participantes eram idosos. Eles e suas famílias tinham ansiedade sobre a condução de veículos. Os 66 participantes (44 homens e 22 mulheres) foram divididos em grupos com e sem experiência de acidente de trânsito, e foram examinados os seguintes testes: testes cognitivos gerais e funções executivas, o teste NPA, e um Teste de Reação de Extremidade Inferior original. Cada participante foi solicitado a pressionar o pedal de “freio” ou “acelerador” com o pé direito o mais rápido possível em resposta a uma situação de trânsito mostrada na tela.

**Resultados:**

Comparados aos participantes com reações favoráveis ao Teste de Reação de Extremidade Inferior, aqueles com tempo de reação ruim tenderam a ter mais acidentes de trânsito (OR=6,82) do que o resultado do teste NPA.

**Conclusões:**

s resultados sugerem que a probabilidade de ocorrência de um acidente de trânsito pode ser melhor avaliada por meio do Teste de Reação da Extremidade Inferior.

## INTRODUCTION

Traffic accidents by older drivers are a social urgent problem and there is a need to develop reliable measures of driving ability.

People with mild cognitive impairment (MCI) or early dementia show driving disability. Di et al.^1^ used machine learning to predict incident MCI and dementia using monthly driving data from in-vehicle recording devices. Babulal et al.^2^ examined whether driving behavior can predict preclinical Alzheimer’s disease (AD). Driving can be used as a novel neurobehavioral marker to identify the presence of preclinical AD. A couple of systematic reviews concluded that all cognitive domains apart from language reported to show a moderate association with on-road driving outcome in mild dementia^3^, we are still far from a widely accepted approach of driving ability evaluation in this increasing population^4^.

However, a real-world driving test cannot be performed in Japan for license testing in all drivers, although driving simulation and neuropsychological tests are used for evaluating driving ability.

The National Police Agency (NPA) in Japan has standardized a *Cognitive Function Test* (NPA test) for renewal of a driver’s license for adults aged ≥75 years. It consists of time orientation, figure naming and recall after interference, and clock drawing. When a driver is identified as Class 1 (suspected decrease of cognitive function), they have to submit a medical certificate. The guidelines require evaluations of dementia, such as AD or vascular dementia; higher brain functions including aphasia, apraxia, visuospatial function, and executive function; and the Clinical Dementia Rating (CDR)^3,4^ and suspected MCI. However, we think driving ability cannot be evaluated simply by testing of cognitive function based on the belief that these abilities are equivalent, as in the NPA test.

In driving a car, it is important to use the right foot in different ways to control the accelerator and brake pedals. These pedals have conflicting functions and are located in a place where a driver cannot confirm the pedals visually. The upper extremities are used to control the wheel, but even if a driver visually confirms a risk and intends to control the wheel, the risk cannot be avoided without appropriate use of the right foot.

For example, the Trail-Making Test (TMT) is a well-known measure of executive function, but a version of this test for the foot is not common^5^. The nerves of the foot are located furthest from the brain and are easily affected by aging. In patients with cerebrovascular disease, vascular Parkinsonism may be present even if there is no clear decrease in cognitive functions that control use of the upper extremities and language. AD with cerebrovascular disease is the main form of dementia in Japan, followed by vascular dementia^6,7^. Thus, many people with very mild symptoms may be living in the community.

This background raises the question of the best test for detection of reduced function of the lower extremities for driving. The Posner paradigm is used as a visual attention task for the upper extremities^8,9^. This is a simple test, in which an examinee is requested to push the right or left button on a computer screen when a light is shown on the right or left side with hands, respectively. As a pre-cue, we prepared a valid condition, in which the light is shown in the same place as that in a real test, and an invalid condition. Healthy persons can react to lights on the right and left equally since they suspect a faint when the light is shown on the right side as a pre-cue. Thus, they have no difference in reaction time between the valid and invalid conditions. However, some patients with cognitive dysfunction, especially AD, have a delayed reaction because they are affected by the invalid conditions provided as a pre-cue^9^. This is a disorder of attention shifting, which is a visual attention characteristic.

We thought that this principle may be applicable to a test of the lower extremities. When a red signal is shown, an examinee should press the brake, whereas with a green signal, the examinee should press the accelerator, but should press the brake if a child is seen, even when the light is green. This is a high-level task in which a signal color and a child need to be recognized at the same time to operate the brake or accelerator appropriately. Here, we define this procedure as the “Lower Extremity Reaction Test.” After accumulation of data for accidents in a database of community medicine, we examined the relationships of these data with the results of the NPA test. Our hypothesis was that scores on the Lower Extremity Reaction Test would predict traffic accidents more effectively than those on the NPA test.

## METHODS

### Participants and classification

This was a consecutive outpatient study. The participants were older adults who visited an amnesia clinic in Town A. In Japan, patients who are diagnosed with dementia are required to return their driver licenses, and we use this rule to provide appropriate guidance. The participants had visited the clinic for the first time before diagnosis, at a time when they and their families had anxiety about their driving.

All participants were older adults who had got driving licenses before 40 years or more, and they all drive their cars 1–2 times a week. All participants were assessed by a neurologist (K.M.) for the functional capacity in lower limbs, and no participants revealed weakness of muscle strength, sensory disturbance, and coordination problem. Their ADL levels were good and did not have orthopedic problems.

### Inclusion criteria

The participants were required to have a driver’s license and to have driven their cars in the local area over the past 2 years. Since they had passed the test for renewal of the driver license, other than the NPA test, we judged that they had no problem with near visual acuity.

### Exclusion criteria

Older adults with paralysis or sensory deficit confirmed in a neurological test were excluded.

Older adults who take dementia medication such as a cholinesterase inhibitor, a drug for improvement of cerebral circulation and metabolism, an antiepileptic drug, an antidepressant, or another drug that may affect cerebral circulation and metabolism were excluded from the study.

A total of 66 participants (men: 44, women: 22) were divided into groups with and without experience of a traffic accident (the accident group vs. the nonaccident group), and correlations with the following test results were examined.

### Ethical considerations

Written informed consent was obtained from all participants and their families before the study was conducted. The study was performed after obtaining approval from the Ethical Committee of Tohoku University School of Medicine.

### Tests conducted in the study

#### Questionnaire survey on traffic accidents

Traffic accidents, including property damage accidents, were evaluated based on self-reporting and information from families of the participants. According to the Japanese Road Traffic Act 2, traffic accidents are defined as accidents causing injury (or death) or property damage accidents.

##### Neuropsychological tests

###### General cognitive function and executive function test

The Mini-Mental State Examination (MMSE) was used as a general cognitive function test, and the TMT-A and Digit Symbol (DS) test (120 s) were used as executive function tests.

###### National police agency test

The NPA test was performed to categorize the participants into Classes 1, 2, and 3. Class 1 shows suspected decrease of cognitive function (grossly correspond to dementia), Class 2 indicates possible decrease of cognitive function (grossly correspond to MCI), and Class 3 notes no cognitive impairment.

##### Original lower extremity reaction test

**Figure 1.**
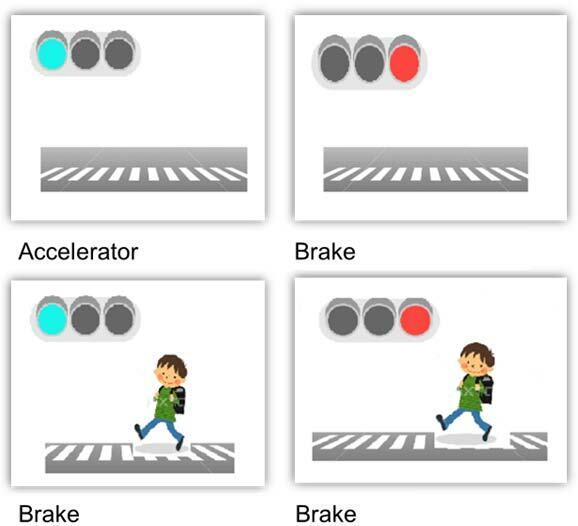
A computer designed for persons with a disability of the upper extremities was used, since this has two pedals placed side by side for use with the lower extremities. Each participant sitting position was asked to press the “brake” or “accelerator” pedal as quickly as possible in response to a traffic situation indicated by each of four images shown on the screen.

The Posner paradigm as a visual attention task^8,9^ for the upper extremities was adapted to the lower extremities. A computer designed for persons with a disability of the upper extremities was used, since this has two pedals placed side by side for use with the lower extremities. Each participant was asked to press the “brake” or “accelerator” pedal as quickly as possible in response to a traffic situation indicated by each of four images shown on the screen (Figure 1).

The reaction times of the lower extremity were measured and correct and incorrect reactions were recorded. The following information was given to the participants:

- Pedals are positioned on the right and left sides under a desk with a computer screen, as the “accelerator” and “brake,” respectively. The pedals under the desk cannot be seen.
- Only the right foot should be used to push the accelerator or the brake.
- After a red or green light is shown on the computer screen, the brake or accelerator should be pressed as quickly as possible, using the right foot (simple condition).
- A more complex condition will be used to simulate a driving situation as closely as possible. For example, if a child is seen, even when the light is green, the brake should be pressed.

### Statistical analyses

#### Demographics

The demographics (together with the results of Analysis 1) of the two groups are summarized.

#### Relationships between the lower extremity reaction test and others

To examine how the results of the Lower Extremity Reaction Test are related to function, the number of correct actions and average reaction time (s) were defined as objective variables. Spearman rank-correlation coefficients were then calculated for these variables with age, scores on the MMSE, TMT-A, DS, and NPA tests.

#### Difference between the accident group and the nonaccident group

The test scores are compared between the accident group and the nonaccident group. The Mann-Whitney U test was performed for both groups without logistic analysis.

Prediction of traffic accidents using the lower extremity reaction test and the National Police Agency test

A crossover analysis was performed to examine the relationship of traffic accidents based on the classification using the NPA test and the reaction time measured under complex conditions in the Lower Extremity Reaction Test, using a chi-square test and calculation of the odds ratio.

Logistic analysis of traffic accidents was also performed, using all measures as forced entry, and that of the number of correct actions and reaction time as explanatory variables.

The SPSS software was used for statistical analysis.

## RESULTS

### Demographics

The demographics (together with the results of Analysis 1, see below) of the two groups are shown in Table 1.

**Table 1.** Demographics and differences between accident and nonaccident groups.

		Accident	Nonaccident	t-test, chi-square test, Mann-Whitney U test	p-value
Participants (men/women)		32 (24/8)	36 (20/16)	2.8	0.94^*^
Age (years)		77.6 (5.9)	75.2 (7.0)	1.5	0.14^†^
Education (years)		11.3 (2.3)	11.6 (2.5)	-0.56	0.58^†^
MMSE (score)		19.1 (5.7)	20.5 (6.2)	328.0	0.45
Trail-making test, s		81.0 (34.4)	66.0 (38.4)	208.0	0.046
Digit Symbol (120 s)		32.5 (13.6)	48.3 (18.3)	37.0	0.045
NPA test: Class I/Class II/Class III		16/12/4	13/13/10	2.7	0.26^*^
Lower extremity reaction test	Correction	23.6 (8.2)	27.9 (3.6)	363.5	0.008
	Reaction time	1.17 (0.41)	1.03 (0.66)	351.0	0.006

Chi-square test. ^†^t-test. MMSE: Mini-Mental State Examination; NPA: National Police Agency.

There was no significant difference in age, years of education, and total score on the MMSE^10^ between the groups.

### Relationships between the lower extremity reaction test and other tests

Table 2 shows the results of Analysis 1.

**Table 2.** Relationships between the lower extremity reaction test and other tests.

	Number of correct actions		Average reaction time	
	rs	p-value	rs	p-value
Age	-0.30	0.012	0.17	0.170
NPA test	0.34	0.005	-0.55	<0.001
MMSE	0.54	<0.001	-0.52	<0.001
Trail-making test A	-0.49	<0.001	0.35	0.013
Digit symbol (120 s)	0.63	<0.001	-0.68	<0.001

NPA: National Police Agency; MMSE: Mini-Mental State Examination.

### Difference between the accident group and nonaccident group

Table 1 shows the results of Analysis 2.

### Prediction of traffic accidents using the lower extremity reaction test and the National Police Agency test

**Figure 2.**
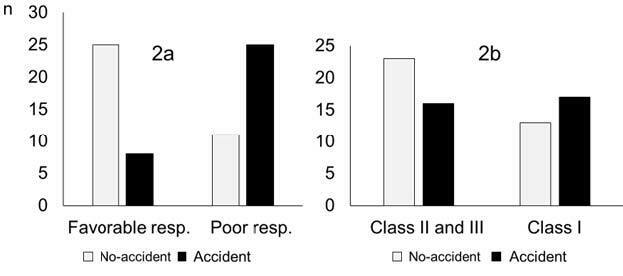
Participants with a poor reaction time had experienced more traffic accidents, compared to those with a favorable reaction [chi-square value=9.89, p=0.017 (two-sided), odds ratio (OR)=5.00]. **A.** The relationship between reaction time and experience of traffic accidents for participants with correct reactions. Compared to participants with favorable reactions, those with poor reaction time tended to have more traffic accidents [chi-square=13.40, p=0.0003 (two-sided), OR=6.82]. **B.** The results for the NPA test showed that older participants in Class 1 also tended to have traffic accidents more than those in Classes 2 and 3 [chi-square=1.7, p=0.20 (two-sided), OR=1.88]; however, the Lower Extremity Reaction Test gave a higher odds ratio.

Figure 2 illustrates the results of Analysis 3.

Participants with a poor reaction time had experienced more traffic accidents, compared to those with a favorable reaction. The relationship between reaction time and experience of traffic accidents for participants with correct reactions is shown in Figure 2A. Compared to participants with favorable reactions, those with poor reaction time tended to have more traffic accidents. The results for the NPA test (Figure 2B) showed that older participants in Class 1 also tended to have traffic accidents more than those in Classes 2 and 3; however, the Lower Extremity Reaction Test gave a higher odds ratio.

The results of logistic analysis forced entry of all measures showed all negative findings. However, by focusing the number of correct reactions and the reaction time as variables, only the number of correct reactions had a significant correlation with the probability of having a traffic accident. We have entered all measures shown in Table 1.

## DISCUSSION

### Summary of results

We developed an original Lower Extremity Reaction Test for asking participants to press the “brake” or “accelerator” pedal by the right foot as quickly as possible in response to a traffic situation shown on the screen. Compared to participants with favorable reactions to the Lower Extremity Reaction Test, those with poor reaction time tended to have more traffic accidents, rather than the result of the NPA test.

### Relationships between the lower extremity reaction test and other tests

The results for the Lower Extremity Reaction Test were correlated with general and executive function, which suggests a high validity of the test. There was no correlation with age, which indicates that the results of the test are not affected by visual acuity or motor response, which are normally decreased by aging.

### Markers of traffic accidents: National Police Agency test versus lower extremity reaction

Participants with experience of a traffic accident had a significantly lower average reaction time(s) on the Lower Extremity Reaction Test, even after multivariate adjustment. The odds ratios suggest that the probability of having a traffic accident can be partially predicted by a neuropsychological test with use of the upper extremities, but that this probability cannot be fully determined without evaluation of use of the right foot for pressing the accelerator or brake.

### Pedal operation

In a study by Hasegawa et al.^11^, participants using a driving simulator were required to stop a vehicle as quickly as possible when a red signal was presented on a monitor. In most trials, the vehicle stopped when the brake pedal was applied in a normal manner. In a few trials, however, stepping on the brake pedal resulted in sudden acceleration of the vehicle (unintended acceleration). These results suggest that there are age-related differences in error detection and correction abilities in unexpected situations, due to incorrect pedal manipulation. During a situation of unintended acceleration, the ability to correct pedal stepping declined in older subjects; however, there was no significant age-related decline in the quickness of performing regular and simple pedal stepping.

### Neurological basis of safe driving

A recent review^12^ indicated that widespread brain networks, including the occipital, parietal, frontal, and cerebellar regions, are required for safe driving. These networks are vulnerable in AD pathology that shows extensive neocortical brain damage, and early pathological changes in the posterior temporo-parietal regions are responsible for impaired driving in the early stage of AD. Using a driving simulator and functional magnetic resonance imaging, Choi et al.^12^ determined the overall effective connectivity between brain areas related to driving. In both hemispheres, visual attention, inhibitory control movement, and episodic memory retrieval pathways were prominent. The activation of these pathways indicates that driving requires multidomain executive function, in addition to vision. Moreover, pathway activation is influenced by driving experience and familiarity of the driver. An interesting finding of Choi et al. was the prominence of the inhibitory control movement pathway in both hemispheres^12^. Research on inhibitory control has mainly been conducted using go/no-go tasks^13^, and there are no reports associated with driving. Inhibitory control is a multidomain executive function critical for flexible responsivity to changing task demands and thus is an essential component of adaptive behavioral regulation.

### Limitations

The participants in this study were patients who visited an amnesia clinic for the first time; therefore, there is a possibility that patients with dementia were included. Generally speaking, older drivers in Japan do not want to be diagnosed by medical doctors, since their driving licenses should be returned to the police office once diagnosed with dementia. Our participants had also this behavior and not all of them had medical diagnosis. This is a limitation of this study and had added a description of these in the revise manuscript. In the future, it will be required to perform tests for older adults classified as healthy (CDR 0) or with possible dementia (CDR 0.5), as determined by using CDR evaluation. This study also includes the first use of the Posner cueing task for the lower extremities, and there are no standard values for this test. Results for CDR 0 subjects would provide these values.
